# PROFILE OF PATIENTS WHO SEEK THE BARIATRIC SURGERY

**DOI:** 10.1590/S0102-6720201500040013

**Published:** 2015

**Authors:** Paola Turchiello da SILVA, Luciana Dapieve PATIAS, Glauco da Costa ALVAREZ, Vanessa Ramos KIRSTEN, Elisângela COLPO, Cristina Machado Bragança de MORAES

**Affiliations:** Surgery Clinic of Obesity and Digestive System of Santa Maria, Santa Maria, RS, Brazil

**Keywords:** Obesity, Bariatric surgery, Epidemiology

## Abstract

***Background*:**

Nowadays obesity is a chronic disease considered one of the greatest problems in public healthy. Showing to be effective in a short and long term, the bariatric surgery has emerged as an optional treatment for morbid obesity.

**Aim:**

Identify the profile of patients seeking bariatric surgery.

**Methods:**

Were interviewed 100 patients in preoperative nutritional monitoring of bariatric surgery. The study was conducted by applying a questionnaire prepared according to the research objectives.

**Results:**

From the individuals that were seeking bariatric surgery, 78% were female, 62% were married and 69% reported physical activity. The average age of those surveyed was 37±10.83 years and mean body mass index (BMI) was 43.51± 6.25 kg/m². The comorbidity more prevalent in this group was high blood pressure (51%). In previous treatments for weight reduction, 92% have already done hypocaloric diet followed by anorectic drug (83%). The success of these treatments was reported by 92% of patients; however, the weight lost was recovered in less than one year of 75%. Patients with diabetes mellitus and dyslipidemia had higher BMI values. The patients with comorbidities showed lower levels of BMI.

**Conclusion:**

The profile of patients who sought surgical treatment for their obesity were predominantly women with a family background of obesity and obesity-related comorbidities, especially hypertension and diabetes mellitus.

## Abstract (pt)

**Racional:**

A obesidade é doença crônica considerada atualmente um dos maiores problemas de saúde pública. Mostrando-se eficaz em curto e longo prazo a cirurgia bariátrica surgiu como opção de tratamento para a obesidade grau III.

**Objetivo:**

Identificar o perfil dos pacientes que procuram a cirurgia bariátrica.

**Métodos:**

Foram entrevistados 100 pacientes em acompanhamento nutricional pré-operatório de cirurgia bariátrica. O estudo foi realizado através da aplicação de um questionário elaborado de acordo com os objetivos da pesquisa.

**Resultados:**

Dos indivíduos que buscaram a cirurgia bariátrica, 78% eram do sexo feminino, 62% casados e 69% dos entrevistados afirmaram praticar atividade física. A média de idade dos indivíduos pesquisados foi de 37±10,83 anos e a média de IMC foi de 43,51±6,25 kg/m². A comorbidade que mais prevaleceu no grupo pesquisado foi a hipertensão arterial (51%). Dos tratamentos prévios para redução de peso, 92% já realizou dieta hipocalórica seguido de medicamento anorexígeno (83%). O sucesso destes tratamentos foi relatado por 92% dos pacientes; porém, o peso perdido foi recuperado em menos de um ano por 75%. Ao associarem-se as comorbidades com o IMC, encontrou-se diferença significativa entre os pacientes com e sem diabete melito, assim como os com e sem dislipidemia. Os pacientes que apresentavam comorbidades mostraram menores níveis de IMC.

**Conclusão:**

O perfil dos pacientes que buscaram correção cirúrgica para sua obesidade eram predominantemente mulheres, com histórico familiar de obesidade, com média de idade de 37 anos e com comorbidades relacionadas à obesidade, com destaque para hipertensão arterial e diabete melito.

## INTRODUCTION

Nowadays, obesity is considered one of the biggest problems in public health^4^. Seen as a worldwide epidemic, it defined as the accumulation of fat tissue in the organism, result of the energy intake that is over the expenditure energy^11^.

According to the Pesquisa de Orçamentos Familiares de 2008-2009^10^, 50% of men and 48% of women were overweight, from these group 12,5% of men and 16,9% of women were obese.

The risk of medical comorbidity is directly associated to the Body Mass Index (BMI), the abdominal fat or visceral is an independent risk factor to diseases related to overweight and obesity^20^.

Treatments used to treat morbid obese patients, pharmacologic and dietetic, have low prevalence, mainly because there is not a changing in life style. The success, depends of the constantly vigilance of food intake - beyond factors as familiar and social support, self monitoring and most of the time, they are no well performed, creating disappointment among patients^7^.

Surgery treatments have been efficient in a short and long term, by weight loss and solving the medical comorbidity for treatments of grade II obesity^16^. Some requirements have to be followed to indicate the surgery, as BMI equal or more than 40 kg/m² without medical comorbidity associated or more than 35 kg/m² with medical comorbidity^3^. 

This study is aimed to identify the patient´s profile that seek the bariatric surgery.

## METHOD

This study has been reviewed and approved by the Centro Universitário Franciscano Internal Review Board (No. 235.073) and informed consent was obtained from all participants. 

Were interviewed 100 patients at the Surgery Clinic of Obesity and Digestive System of Santa Maria, Santa Maria, RS, Brazil during the nutritional assessment of the pre-bariatric surgery in the period of April to May, 2013.

The study was conducted through a questionnaire with personal information (gender, age, marital status, profession) and specific information about their clinic obesity history, physical activity, alcohol intake, smoking and others method to weight loss previously to the bariatric surgery. It was also asked the motivation to perform the bariatric surgery as a treatment and what would be the main goal to their weight loss.

Data were analyzed in the SPSS version 18.0 and presented as a simple descriptive statistic (mean±SD and percentage). Comparison between the means was done using t-Student test.

## RESULTS

From the subjects submitted to bariatric surgery, the sample was characterized mainly by women (78%), married (62%), physically active (69%) and low prevalence of smokers (11%) and alcohol drinkers (10%) (Table 1). 

**TABLE 1:** - Demographic profile, alcohol intake, smoking and physical activity practice in subjects that seek the bariatric surgery (2013)

	**Gender**	**Marital Status**	**Smokin**	**Alchoholic Beberage**	**Physical Activity Practice**					
	**M**	**F**	**Mar**	**Sin**	**Yes**	**No**	**Yes**	**No**	**Yes**	**No**
%	22	78	38	62	11	89	10	90	69	31
(n)	(22)	(78)	(38)	(62)	(11)	(89)	(10)	(90)	(69)	(31)

**FIGURE 1:**
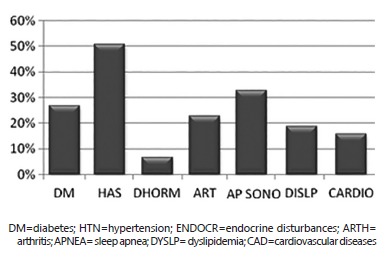
- Prevalence of medical comorbidity associated to obesity in patients submitted to bariatric surgery, Santa Maria-RS (2013)

The average age of the subject analyzed was of 37.8±10.8 years old (minimum age of 17 and maximum 68 years old). Regarding the medical comorbidity 24% did not report, 39% reported between one and two and 37% reported three. Among them, the most frequent were hypertension (51%), apnea (33%) and diabetes (27%) (Figure 1). 

Regarding the nutritional status classification, 81% were classified as obesity grade III. Almost all subjects (83%), reported an obesity family background, 54% were overweight since childhood (Table 2). 

**TABLE 2:** - Nutritional status, family background and childhood overweight

	**Nutritional status**	**Family background**	**Overweight in childhood**			
	**Obes II**	**Obes III**	**Yes**	**No**	**Yes**	**No**
%	19	81	83	17	54	46
(n)	(19)	(81)	(83)	(17)	(54)	(46)

The average BMI was 43.51±6.25 kg/m², being the lowest value found 35 kg/m² and the highest 85.78 kg/m².

**FIGURE 2:**
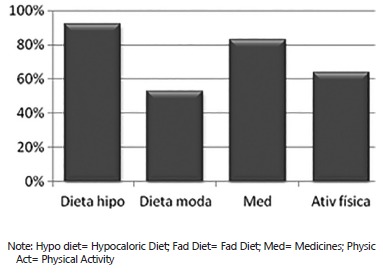
- Previous methods to weight loss

When the subjects were asked regarding the use of others previous treatment to the weight loss, 92% answered that they have already done hypocaloric diet and 53% the fad diet as a treatment. The use of appetite suppressants were appointed by a high number of subjects (83%) (Figure 2).

The success of the treatments highlighted in the picture 2 was reported by 92% of the patients; however the weight was regained in less than one year by 75% of the interviewees. 

Concerning the healthcare professional's supervision to weight loss treatment, 24% have been supervised by nutritionists; 20% by a physician; 42% by both; and 14% without professional supervision. 

When comparing the BMI with gender and marital status, men have showed a higher BMI than women, as well as it was higher in single individuals (p<0,01).

**TABLE 3:** - Relation between BMI, gender and marital status

	**BMI vs Gender**	**BMI vs Marital status**				
	**Female**	**Male**	**p**	**Single**	**Married**	**p**
BMI (kg/m²)	42.04 ±4.45	48.04 ±9.82	<0,01	44.12 ±5.1	42.89 ±7.21	<0,01

Results are expressed as mean±SD (*) t-test

When associate BMI with medical comorbidities, it was verified that patients without diabetes and dyslipidemia have showed lower average values to the BMI (p <0,05) (Table 4). 

**TABLE 4:** - Average BMI value comparison with medical comorbidities presence

**Medical comorbidities**		**BMI (kg/m²)±SD**	**p**
Diabetes	Yes	41.25±5.51	0.04
	No	44.14±6.68	
Hypertension	Yes	43.44±7.57	0.89
	No	43.26±5.2	
Apnea	Yes	44.04±9.26	0.45
	No	43.02±4.61	
Hormonal diseases	Yes	41.22±4.13	0.36
	No	43.51±6.62	
Arthritis	Yes	42.55±4.40	0.50
	No	43.59±6.99	
Dyslipidemia	Yes	39.98±5.59	0.01
	No	44.15±6.46	
Cardio	Yes	44.01±12.42	0.66
	No	43.23±4.71	

**FIGURE 3:**
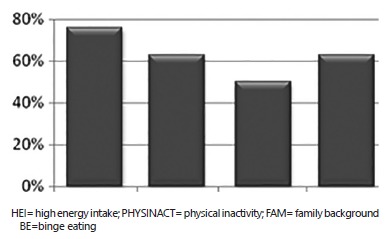
- Factors associated to overweight

The factors that most influenced in the weight gain were: high energy intake (76%), physical inactivity (63%), family background (50%) and binge eating (63%). The most part of the sample pointed more than one factor (Figure 3). 

The main reasons that motivated the patients to be submitted to bariatric surgery as a treatment, 49% pointed the failure in the previous treatments, 39% medical comorbidities, 12% by the treatment efficiency and facility in losing weight.

The main objective to be submitted to bariatric surgery, 87% reported the improvement in the life quality; 82% the improvement in their health; 34% the beauty factor; and 15% to be accepted in society.

## DISCUSSION

The main findings of this study demonstrate that before surgery, the patients were mainly women, with high prevalence of medical comorbidities, showing a family obesity history and the performance of previous dietetics interventions without any success. Regarding the age, there is a trend to this type of surgery, it has increasingly been performed in young patients with a high level of obesity, as also highlighted by others authors^19^^,^ 8.

According to Craig and Trusweel, the marriage can influence the weight gain, mainly between women. The reasons could be the decrease of energy expenditure and alteration of eating habits^5^. In this study, differently, the BMI has been higher in single patients, and men had higher BMI than women. These results can be justified by the social life of single people associated to habits as high alcohol intake and a great number of out-of-home-eating.

Regarding the medical comorbidities, 76% pointed some disease, data higher than the found by Cambi et al., which only 40% of patients had it^2^. According to the World Health Organization, as higher as the value of BMI, there is an increase the risk of medical comorbidities, predominating hypertension, followed by apnea and diabetes^15^. Lichtblau et al. found different results with prevalence of respiratory problems (70%) and osteoarticular (63,3%), followed by hypertension (53,3%)^12^.

Concerning the smoking and drinking, it has been found a low consumption that is considered satisfactory according to Still, Benotti, Wood et al. the alcohol dependency or illicit drugs is contraindicated to the bariatric surgery^17^.

In this study the high number of physical activity practitioners was probably because of the multidisciplinary group supervision in the preoperative phase 13.

Although the pharmacological treatment helps the weight loss in patients, the effectiveness and medicine safety for longer than two years is not completely established. Success in previous treatments before the surgery, was highlighted by 92% of patients; however, the weight was regained in less than one year by 75% of the patients. 

The National Institute of Health, estimate that almost 80% of people that lose weight tend to regain it, and 1/3 to 2/3 of this recovery occurs just in the first year after losing^14^. 

The biggest part of the subjects studied has reported a family obesity history and overweight since childhood, supporting the data found by Porto et al.^15^. It is highly probable that the polygenic inheritance is a determinant factor. The risk of being obese in childhood, can increase when parents are also obese. When none of the parents are obese, the risk is of 9%, however when one parent is obese, it increases to 50% and to 80% when both are obese^18^. 

Most the causes of obesity are not easily identified. It is multifactorial and can be classified into two major contexts: exogenous, influenced by external factors of behavioral origin, dietary and environmental in 95% of cases, and endogenous, by genetic, neuropsychological, endocrinology and metabolism at 5% 6.

In a previous study it was demonstrated that binge eating is present in 27-47% patients that sought for the bariatric surgery to weight loss^1^. 

Surgical treatment promotes the weight loss, improves metabolism and life quality, according to the main goal sought by the patients of this study that were submitted to bariatric surgery^9^. 

## CONCLUSION

The profile of patients who sought surgical treatment for their obesity were predominantly women with a family background of obesity and obesity-related comorbidities, especially hypertension and diabetes mellitus. 
